# Radio Frequency Identification and Sensing Techniques and Their Applications—A Review of the State-of-the-Art

**DOI:** 10.3390/s19184012

**Published:** 2019-09-17

**Authors:** Lei Cui, Zonghua Zhang, Nan Gao, Zhaozong Meng, Zhen Li

**Affiliations:** 1 School of Mechanical Engineering, Hebei University of Technology, Tianjin 300130, China; lc.cui@outlook.com (L.C.); zhzhang@hebut.edu.cn (Z.Z.); ngao@hebut.edu.cn (N.G.); 2 College of Automation Engineering, Nanjing University of Aeronautics and Astronautics, Nanjing 211106, China; zhenli@nuaa.edu.cn; 3 Nondestructive Detection and Monitoring Technology for High Speed Transportation Facilities, Key Laboratory of Ministry of Industry and Information Technology, Nanjing 211106, China

**Keywords:** radio frequency energy harvesting, radio frequency identification (RFID), RFID sensor, inductive coupling, backscattering

## Abstract

Radio Frequency Identification (RFID) sensors, integrating the features of Wireless Information and Power Transfer (WIPT), object identification and energy efficient sensing capabilities, have been considered a new paradigm of sensing and communication for the futuristic information systems. RFID sensor tags featuring contactless sensing, wireless information transfer, wireless powered, light weight, non-line-of-sight transmission, flexible and pasteable are a critical enabling technology for future Internet-of-Things (IoT) applications, such as manufacturing, logistics, healthcare, agriculture and food. They have attracted numerous research efforts due to their innovative potential in the various application fields. However, there has been a gap between the in-lab investigations and the practical IoT application scenarios, which has motivated this survey of this research to identify the promising enabling techniques and the underlying challenges. This study aims to provide an exhaustive review on the state-of-art RFID sensor technologies from the system implementation perspective by focusing on the fundamental RF energy harvesting theories, the recent technical progresses and commercial solutions, innovative applications and some RFID sensor based IoT solutions, identify the underlying technological challenges at the time being, and give the future research trends and promising application fields in the rich sensing applications of the forthcoming IoT era.

## 1. Introduction

Radio Frequency Identification (RFID) technology, as a key enabling technique of the Internet of Things (IoT) sensing layer now finds applications in a wide spectrum of fields for data integration and management, including human identification, logistics and retail, access control, parking management, indoor localization, etc. [1,2,3]. Compared to the other alternatives, such as barcodes and QR codes, the radio frequency-powered identification approach featuring contactless, wireless powered, non-line-of-sight, read- and writeable, light weight, and multiple tag simultaneous reading allows the connected “things” to be identifiable for further data communication and integration [4,5]. The functions of RFID are to collect RF energy from the interrogator with its antenna, either Ultra-high Frequency (UHF) or High-Frequency (HF), activate the RFID chip in the tag, and transmit an ID code back to the interrogator, where the ID code is a fixed number used as a unique identifier of a “thing”. The features of the “things” corresponding to their identities can be saved into a database and updated in real-time for data management to meet the needs of different applications.

However, since the RFID chip is an Integrated Circuit (IC) powered by RF energy, components with sensing capability can also be potentially integrated into RFID tags for simultaneous identification and sensing purposes. The integration of RFID tags with sensing components could eventually deliver identification and sensing capability in a wireless powered, contactless, and non-line-of-sight way. Simultaneous Wireless Information and Power Transfer (SWIPT), different from the conventional wired- or battery-powered sensing [6,7], has become a new paradigm of sensing and communication, which could potentially reshape the future rich-sensing IoT world [8]. On the one hand, due to the wide coverage and mobility of RFID interrogators, the measurement of RFID sensor tagged “things” is no longer limited to specific locations. On the other hand, since RFID sensors can be fully passive, there is no need to frequently change the batteries of the RFID sensors like in traditional wireless sensor nodes. Therefore, the information sensing procedure of RFID sensors becomes much more flexible and convenient, and its applications can be extended into a wider field. 

Due to the convenience of RFID sensing techniques, they have attracted many research efforts in the recent years and plenty of exemplary novel applications can be found in the literature. Firstly, they have been widely applied in industry for the sensing of temperature and humidity [9], strain [10], pressure [11], steel corrosion and cracks [12], concrete structure [13], pipeline integrity monitoring [14], etc. Secondly, they are also popular in healthcare, in devices such as wearable and implanted sensing devices for glucose monitoring [15], blood pressure [16], intraocular pressure [17], and on-skin monitoring discrimination of breath anomalies [18] etc. The above examples are some exemplary cases of investigations, and the research and applications are not limited to them [19]. The investigations of RFID sensing techniques can be mainly divided into four categories: (1) radio frequency energy harvesting efficiency; (2) the integration of RFID and sensing techniques; (3) chip-less RFID techniques; (4) RFID sensor network technology.

Although significant technical progress regarding the research and application of RFID sensing techniques has already been achieved, there is still a gap between in-lab investigations and practical applications. In addition, there are also technical issues in the integration of RFID technology with sensing components that have not been sufficiently addressed. A comprehensive report summarizing the recent technical progress and challenges of the state-of-the-art in the literature is also lacking. The above reasons have motivated this review work to report the state-of-the-art in RFID sensor technical solutions, the recent technical progress, the technical challenges, and to forecast the direction of future investigations in the rich sensing applications of the forthcoming IoT era.

The structure of this article is organized as follows: Firstly, Section 2 presents the fundamentals of RF energy harvesting, RFID, and RFID sensing; secondly, Section 3 gives the recent technical progress of RFID sensing techniques, including RF energy harvesting efficiency, the integration of RFID with sensors, the commercial solutions and innovative applications in both academia and industry. Then, Section 4 identifies the potential challenges in this particular field, followed by Section 5 which presents an outlook of the future perspectives of novel RFID applications. Finally, Section 6 concludes the work of this investigation.

## 2. The Fundamentals of RF Energy Harvesting and RFID Sensor Techniques 

The source power of energy harvesting can be in different forms, such as mechanical movement and vibrations, solar energy, thermal energy, electromagnetic power, etc. Since the early 1990s, when Tesla and Hertz proposed the concept of wireless energy harvesting [20,21], research on wireless energy collection has become more and more extensive in the scientific community. According to the fundamentals of RFID, radio frequency energy harvesting can be divided into inductive coupling and backscattering [22]. The HF RFID working at the carrier frequency 13.56 MHz transmits and receives power with near-field inductive coupling [23], and the UHF working at the carrier frequency 840–960 MHz deals with power transmission and reception with far-field backscattering [24]. In addition, chip-less RFID, which performs its measurements using the shift resonant frequency has also become a focal research topic. This investigation focuses on the HF and UHF RFID sensor techniques, for which, new investigations and novel technical solutions have been widely reported in the recent few years.

### 2.1. HF Inductive Coupling and HF RFID Sensor Techniques

**Figure 1:**
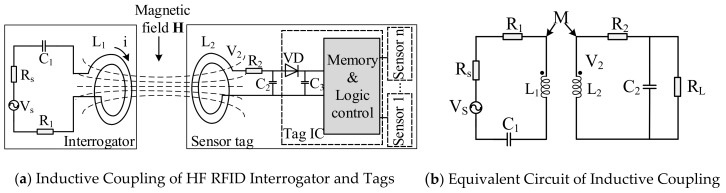
The Fundamentals of HF RFID Sensors.

HF RFID sensor devices transmit or receive both energy and data through inductive coupling, which is achieved by the alternating magnetic field between the coil antennas of the interrogator and the tag. The alternating magnetic field is generated by the inductor-capacitor (LC) resonant tank, the structure and the equivalent circuits of which are as depicted in Figure 1 [25,26].

As shown in Figure 1, *V_s_* is the RF source in the interrogator, *L_1_* and *C_1_* constitute a resonant tank (resonance to the frequency *V_s_*) for power transmission, and the resonant frequency is *f_0_*, normally 13.56 MHz for HF RFID. The tag antenna *L_2_* and *C_2_* constitute the receiver, which works at the same frequency. The resonant frequency can be calculated as follows:

(1) $$f_{0}=\frac{1}{2\pi \sqrt{L_{1}C_{1}}}=\frac{1}{2\pi \sqrt{L_{2}C_{2}}}$$

The inductive voltage *v_2_* on the tag antenna is directly proportional to the change rate of magnetic flux *Ψ* through the coils, which can be represented by:

(2) $$v_{2}=-\frac{d\Psi }{\mathrm{dt}}=-N_{2}\frac{d\Phi }{\mathrm{dt}}=-N_{2}\frac{d}{\mathrm{dt}}(\int B\cdot \mathrm{dS})=-N_{2}\frac{d}{\mathrm{dt}}\left[\int \frac{\mu_{0}i_{1}a^{2}N_{1}}{2{\left(a^{2}{+r}^{2}\right)}^{3/2}}\cos\alpha \mathrm{dS}\right]$$

where *N_2_* and *N_1_* are the number of windings of the tag and interrogator antenna coils, *Φ* is the magnetic flux of each winding coil, *B* and *S* are the strength of magnetic induction and the area of the coils, *i_1_* is the current of the interrogator coil, *a* is the radius of interrogator coil, *r* is the distance between the two coils, and α is the angle between B and S. In (1), when the angle between B and S is *0⁰*, *v_2_* can be calculated by the following expression:

(3) $$v_{2}=-\left[\frac{\mu_{0}N_{1}N_{2}a^{2}S}{2{\left(a^{2}{+r}^{2}\right)}^{3/2}}\right]\frac{\mathrm{di}}{\mathrm{dt}}=--M\frac{\mathrm{di}}{\mathrm{dt}}$$

where *M* is the mutual induction coefficient, which can be calculated with $M=\frac{\mu_{0}N_{1}N_{2}a^{2}S}{2{\left(a^{2}{+r}^{2}\right)}^{3/2}}$.

Formulas (2) and (3) provide a clear relationship between the power received and the key parameters of the two antennas and their relationship. The voltage *V_2_* can be processed with rectifier and regulator circuits to power the RFID chip and sensor module for data acquisition and information transmission. 

### 2.2. UHF Backscattering and UHF Sensor Techniques

**Figure 2:**
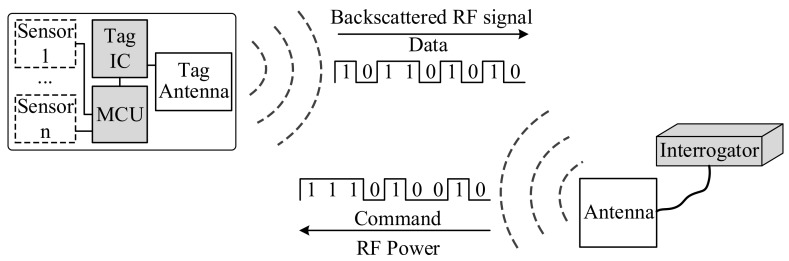
The Fundamentals of UHF RFID Sensors.

Different from a HF RFID system, the operating mode of a UHF RFID sensor device is radar backscattering, consisting of the UHF interrogator, RF transmission path, RFID tag and sensor module, the structure of which is as shown in Figure 2 [27]. The performance of the power transfer is determined by the parameters of the main components, including the radiation power and gain of the transmission antenna and receiving antenna, the wavelength of carrier signal, and the distance between the transmission and receiving antennas.

#### 2.2.1. Interrogator-to-Tag RF Power Transfer

As a passive device, the RFID sensor tag receives the interrogator’s power to complete the sensing and data transmission procedures. According to the mathematical relationship for RF transmission given by Friis equation [28], the power density of RF energy at a distance *R* from the antenna of interrogator *S* can be represented by:

(4) $$S=\frac{P_{T}G_{T}}{{4\pi R}^{2}}$$

where *P_T_* and *G_T_* are the power and gain of the transmission antenna. Suppose the effective dimension of a tag’s receiving antenna is *A_E_* defined as $A_{E}{=\lambda }^{2}G_{\mathrm{Tag}}/(4\pi )$, the gain of the receiving antenna is *G_Tag_*, and the wavelength of RF carrier signal is *λ*, then, the received power *P_Tag_* can be expressed by:

(5) $$P_{\mathrm{Tag}}=S\cdot A_{E}{=P}_{T}{\left(\frac{\lambda }{4\pi R}\right)}^{2}G_{T}G_{\mathrm{Tag}}$$

#### 2.2.2. UHF RFID Power Reflection

The RF power reflected by the tag is directly proportional to the tag’s Radar Cross-Section (RCS) σ. The power reflected by RFID tag *P_Back_* can be denoted with Equation (6), where *S* is the power density, and the power density *S_Back_* can be denoted by Equation (7), *P_T_* and *G_T_* are the transmission power and gain of tag antenna. Suppose *G_R_* is the gain of interrogator antenna, the effective dimension of the receiving antenna is $A_{w}{=\lambda }^{2}G_{R}/\left(4\pi \right)$, and the power received by the interrogator *P_R_* can be represented by Equation (8) [29]:

(6) $$P_{\mathrm{Back}}=S\sigma =\frac{P_{T}G_{T}}{{4\pi R}^{2}}\sigma$$

(7) $$S_{\mathrm{Back}}=\frac{P_{T}G_{T}\sigma }{{\left(4\pi \right)}^{2}R^{4}}$$

(8) $$P_{R}{=S}_{\mathrm{Back}}A_{w}=\frac{P_{T}G_{T}G_{R}\lambda^{2}\sigma }{{\left(4\pi \right)}^{3}R^{4}}$$

The tag’s information, including the tag ID and sensor information, is transmitted to the interrogator by modulating the reflected carrier RF waves. Evidently, the gain of the antennas, the distance between antennas, and the tag’s RCS are key parameters determining the reading distance and efficiency of the resulting RFID sensor measurements.

### 2.3. Chip-less RFID Sensors

**Figure 3:**
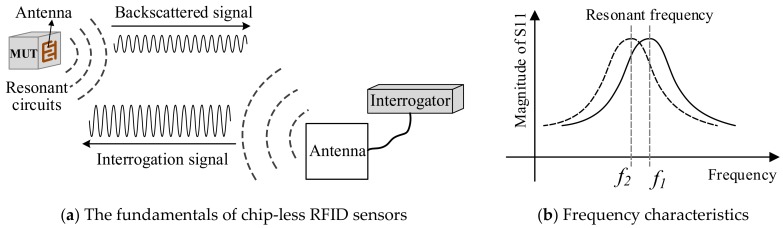
Chip-less RFID Sensors—Fundamentals and Frequency Characteristics.

Chip-less RFID sensors simply change the radar profile of the RFID tags to transmit sensor data without the need for complex integrated circuits, the structure of which is as shown in Figure 3a. When the receiving antenna of the tag receives the signal transmitted by the interrogator, the resonant circuit selects signals of different frequencies to generate a specific resonant frequency and a different resonant amplitude, and then returns the data to the interrogator through the transmitting antenna of the tag. The interrogator then parses the amplitude and phase changes of its specific frequency signal. Finally, the signal ID is encoded according to different spectrum characteristics after data processing [30]. When the chip-less tag is placed on a Material Under Test (MUT), the resonant frequency will drift as the sensor data changes, as shown in Figure 3b [31].

The function of chip-less RFID is determined by the antenna which is normally designed for some particular applications. The ID of chip-less RFIDs are defined by the characteristic frequencies of the tag at a series of specific values [32]. Since some physical features of the materials under test can be reflected by the resonant circuits in the form of capacitance and inductance. The data storage capacity of the chip-less RFIDs is far less than the chipped ones. However, due to silicon chips, the cost of chip-less RFIDs is significantly lower than the chipped ones. Therefore, chip-less RFID has become a competitive choice for low-cost sensing and identification applications [33].

In addition to HF, UHF and chip-less RFID sensors, some applications utilize commercial tags for object distance measurement and localization, which may find use in applications like robot localization and navigation, product localization in large warehouse and logistics, etc.

## 3. RFID Sensor Techniques—Technical Progresses and Innovative Applications

Compared to other environmental energy harvesting approaches, radio frequency is a relatively efficient approach which can obtain more available power in a low-energy environment [34]. In addition, RF energy harvesting integrated with information transmission, termed as Wireless Power Communication (WPC) and SWIPT [35,36], has enabled many innovative applications by the wireless powering, identification and communication. Especially in recent years, with the continuous progress of IoT application techniques [37] and the increase of low-power sensing and micro-controller devices, the integration of sensor techniques with RFID has attracted much research interest and made remarkable progress. This section aims to provide a timely summary of the recent technical progress and innovative applications.

### 3.1. Technical Progress of RFID Integrated Sensing

#### 3.1.1. Novel Antenna Designs: The Front End of RFID Sensing Techniques

**Figure 4:**
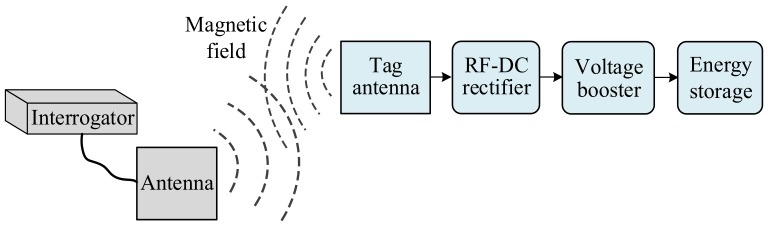
The Diagram of a RF Energy Harvesting Circuit.

The RF energy harvesting circuit is mainly composed of an antenna, a rectifier, a voltage multiplier and an energy storage device as shown in Figure 4, in which the antenna captures the RF energy in the electromagnetic field, then rectifier converts RF energy into a DC voltage through a rectifier, and finally stores the increased voltage of the voltage multiplier in the energy storage device. 

**Figure 5:**
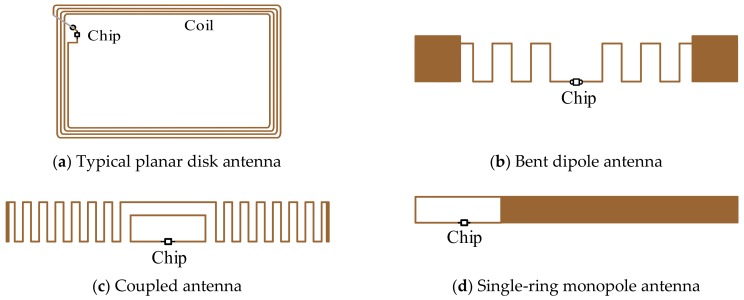
Commonly Used RFID Antennas [38].

The commonly used RFID antennas are shown in Figure 5. The antenna in Figure 5a is normally applied to the HF systems, and the antennas in Figure 5b–d are usually applied to UHF systems. High gain antennas can improve the conversion efficiency and acquire more power, so high gain antennas are preferred. The performance of antennas can be evaluated with parameters including gain, frequency band, radiation pattern, polarization, physical size or fields of application.

Located in the front of a RFID sensing device, the antenna is a key component determining the performance of a RFID sensor, including its reading distance, operation speed and the size of sensor module. Therefore, novel antenna design to enhance the performance of sensor devices and for different application scenarios becomes a hotspot. The main contributions focus on miniaturization of the size, foldable antennas, 3D antennas, metallic surface mountable antennas, multi-band antennas, and omnidirectional and directional antennas, etc., some representatives of which are summarized in Table 1.

With respect to metallic surface mountable antennas, Chen [39] proposed a miniature UHF RFID antenna design for metallic object applications, consisting of two rectangular patches electrically connected through via to the ground plane and a non-connected inter-conductive layer to form an RFID tag antenna. The miniature design is achieved by inserting a conductive layer into the antenna structure to increase the capacitive reactance of the antenna. The overall size is 32 × 18 × 3.2 (mm) and the maximum reading range of RFID tag placed on a metallic object is about 1.5 m. 3D antennas have also become an interest in order to pursue omnidirectional patterns in orientation-independent RFID applications, and many new designs are proposed. Kruesi et al. [43] introduced a miniaturized folded meander line 3D cubic antenna for wireless sensor network (WSN) and RFID application in environmental sensing with a 3D dimension of 3 × 3 × 3 cm, which may find applications in smart packaging by integrating it into a cube’s hollow interior. In addition, multi-band antennas, and omnidirectional and directional antennas are attractive topics as well for different RFID applications. By increasing the working bands of antennas to collect energy over multiple frequency bands, the output voltage of the RF energy harvester can be increased [46]. Keyrouz et al. [47] designed a three-frequency antenna (900 MHz, 1800 MHz, and 2.45 GHz) to collect RF energy, and the energy collection efficiency increased significantly. However, due to the increase in the number of antennas, the dimension of circuit area was enlarged. Liu et al. [50] proposed a capacitively loaded, inductively coupled fed loop antenna with an omnidirectional radiation pattern for UHF RFID tags, with a small size of 43 × 43mm, the measured maximum reading range of the prototype is 9.5 m with a total transmitted power of 4.0 W effective isotropic radiated power (EIRP). The omnidirectional radiation pattern on the horizontal plane makes the proposed tag insensitive to be mounted on different target objects.

Since RFID sensing devices are used for different purposes and application scenarios in different ambient environments, the design of antenna should be customized to the requirements of the particular applications.

**Figure 6:**
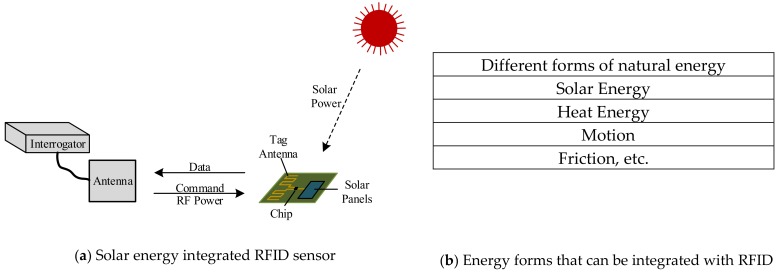
RFID sensor with multi-port energy harvesting.

However, for RFID sensors, the antenna design of a typical RFID tag is versatile and requires the impedance matching for the maximal reading range. But, due to the sensors and various devices, energy consumption has increased significantly. In order to obtain stable RFID sensor data, the RFID sensor antenna can also be configured as a multi-port to collect natural energy to enhance the energy harvesting of the self-powered wireless sensor, as shown in Figure 6.

#### 3.1.2. RF-to-DC Conversion and Power Management

**Figure 7:**
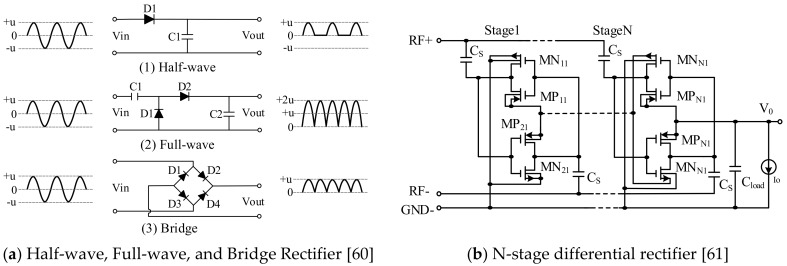
Half-wave, Full-wave, and Bridge Rectifier.

Since the RFID chip and sensing module require DC power for data acquisition and transmission, the RF power needs to be converted to DC to provide the power supply. RF energy harvesting normally uses a multi-stage voltage rectifier to obtain high output voltage [52], and various rectifiers designed to perform the conversion and improve energy harvesting efficiency are presented in [53,54,55,56,57,58,59,60,61]. Half-wave, full-wave, bridge and differential rectification methods, as shown in Figure 7, are the most commonly used solutions [62,63]. Bakhtiar et al. [64] proposed a high sensitivity CMOS rectifier designed with standard threshold voltage devices. The rectifier is designed and laid out in a standard 0.13 μm CMOS process, allowing it to operate even when the input RF power is weak. Ye and Fan [65] describe a high efficiency rectifier circuit for passive UHF RFID applications. The simulation and measurement show efficiency of 30.7% and 15% at low input power level respectively. The rectifier was fabricated in a standard 0.18 μm CMOS process and its core circuit occupied 0.169 × 0.137 mm^2^ silicon area. Ouda et al. [66] proposed a cross-rectifier that can extend the dynamic range of RF power. The rectifier can achieve wireless power supply at different distances, which reduces the problem of reverse leakage without reducing its sensitivity. This design can be used in UHF RFID applications. On-chip measurements show that when the load is 100 kΩ, the sensitivity is −18 dBm at a voltage of 1 V, and the peak conversion efficiency of RF power to DC power is 65%. Yi et al. [53] used a CMOS process in the design of a multi-stage rectifier, which uses a diode with a very low threshold voltage to connect the MOS transistor for energy conversion. However, since a MOS transistor is used, the threshold voltage is increased due to the bulk effect. Liu et al. [54] used a novel diode-connected MOS transistor, and based on this, designed an N-level current rectification circuit, which has higher conversion efficiency and higher output voltage than a rectifier composed of a conventional diode and MOS transistor. However, the threshold voltage of the MOS transistor increases as the usage time increases. Rabén et al. [67] developed a theoretical model for diode-connected MOS transistors with a threshold cancellation technique, and the derived design equations illustrate the tradeoff between the voltage drop and the reverse leakage of the diode. A rectifier was designed and implemented in a 0.35-μm CMOS process, and cadence simulation results of the PCE and the voltage conversion efficiency showed good agreement with the model. Besides UHF, there are also several RF-DC converters operating at 13.56 MHz that can be found in the literature. In [15] and [59], for instance, high-efficiency RF-DC converters exhibiting −4 dBm and 1.2 dBm sensitivity, respectively, are presented. In [68], a HF RF-DC rectifier that uses reverse loss reduction to ensure high output current, even if the DC-DC boost converter limits sensitivity. Colella et al. [69] proposed a four-stage RF-DC converter for HF RFID that is fully integrated using 350 nm CMOS technology. Pelliconi’s two-stage charge pump is used to increase the voltage. Experimental verification showed it can work normally with an RF input power of −19 dBm.

Since the collected RF energy provides the power supply for the operation of the whole RFID sensing module, a voltage multiplier is expected to increase the voltage value and reduce the energy storage time. However, the output voltage of the voltage multiplier changes with process variations and temperature fluctuations (work time is too long or environmental impact). Yuan et al. [70] studied the threshold voltage compensation scheme to improve the effects of voltage multiplier temperature and process variability. Chouhan et al. [71] designed a new cascading method for voltage multiplication circuits, which allows the rectifier to have better power conversion efficiency at lower RF power.

#### 3.1.3. Printable and Flexible RFID Techniques for Sensor Fabrication

**Figure 8:**
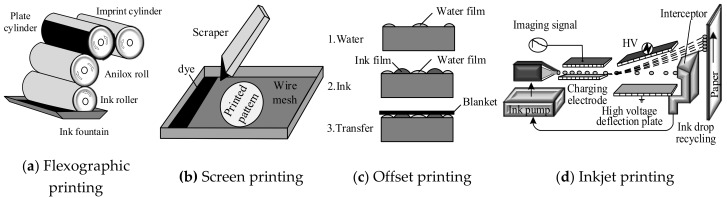
Four Typical Printing Techniques.

Flexibility is an important advantage of RFID tags and RFID sensors, which allows the sensor tags to be able to pasted onto the object under test. The ink-jet printing process has been introduced to the fabrication of RFID sensors [72]. Printed electronics is a new technology that combines traditional printing technology with electronic technology and applies traditional printing technology to electronic manufacturing [73]. Compared with traditional electronic component manufacturing methods, printing technology is more stable in an oxidizing environment, and has the advantages of cost efficient, flexibility, and environmentally friendly. Among them, the contact type mainly includes flexographic printing, offset printing and screen gravure printing, and the non-contact type is mainly inkjet printing. The principle is as shown in Figure 8 [74].

Flexible RFID printing is mainly used to fabricate chip-based RFID antennas and chip-free RFDs, which print nanoparticles such as graphene, silver, or copper on photographic papers, plastics or textiles [75]. In the past ten years, the scarcity of resources, the rapid updating of products and the deteriorating ecological environment have made the market put forward low-cost, flexible and green requirements for modern technology. As an electronic substrate, paper substrates have the advantages of low cost and mass production, which has attracted wide attention [27]. At the same time, although nanoparticle conductive inks are produced in a wide variety of materials (such as copper (Cu), gold (Au), graphene, carbon nanotubes (CNT)), silver nanoparticle ink is the main choice for the electronic conductive trace of printed paper.

**Figure 9:**
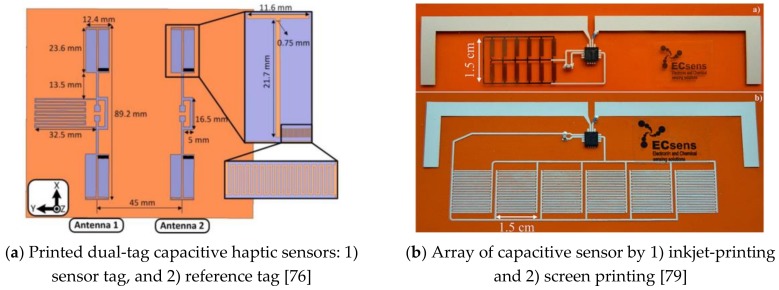
Inkjet Printed RFID Sensors.

Pioneering researchers have already made significant progress regarding RFID applications, and novel investigations can be found in the literature. Paper substrate inkjet-printed RFID sensors have been a prevalent way to fabricate flexible sensors. For example, Kim et al. [27,76] presented a paper substrate silver nanoparticle-based printed sensor fabrication solution and provided two proof-of-concept sensors designs with evaluations: (1) chip-less CNT gas sensor, and (2) UHF dual-tag capacitive haptic sensors (see Figure 9a). Sharif et al. [77] presented a low cost, conductive ink printed small loop integrated with meandered dipole used as an inductive load, which was also connected with RFID chip for metallic can measurement in a smart refrigerator system. Borgese et al. [78] introduced a chip-less RFID humidity sensor based on finite artificial impedance surface (AIS) inkjet printed on a thin sheet of commercial coated paper, composed of three concentric loops thus obtaining three deep and high-Q nulls in the electromagnetic response of the tag. Salmeron et al. [79] reported two printed UHF RFID tags capable of measuring temperature and humidity, as shown in Figure 9b, using the SL900A IC on-chip temperature sensor and capacitive humidity sensor fabricated on polyimide substrate: (1) inkjet-printed array of capacitive humidity sensors, and (2) screen printing interdigitated capacitive sensors.

In the above applications, the printed antennas are connected to an RFID chip and sensing components to allow RF-powered sensing capabilities. The advantages of printed RFID electronics are: (1) sensors can be made flexible by choosing flexible substrates; (2) the cost of RFID sensor fabrication can be effectively reduced; and (3) sensing components can be designed as a printable structure. These advantages allow the RFID sensors to be conveniently fabricated for different investigations and applications with reasonable low cost.

However, the size of the RFID sensor is still a limitation for many practical applications. Since the area of the RFID sensor chip is small, the size of the entire RFID sensor is determined by the size of the antenna. In order to study RFID sensors of small size, the use of textile technology to weave RFID sensors are introduced [80,81,82,83,84,85]. Textile integrated RFID has the advantages of low cost, durability, and the separation of textile and RFID chip manufacturing, making mass production fast. Kalhnayer et al. [80] studied the textile transponder system, in order to achieve a high read/write distance, the antenna uses a textile-based design, but the washability needs to be improved. Vieroth et al. [81] used a flexible substrate on the coupling module package to obtain the flexibility of the package. The test results showed that the package with silver surface finish can withstand all test conditions, even washed at 60 °C. Alonso-González et al. [82] designed a three-layer woven structure that converts the antenna of an RFID tag into a woven type. The label can be processed directly into the garment, making it widely used in the apparel sector. The integration of RFID with textiles is an effective means to obtain the flexibility of RFID sensor devices, which therefore extends the application fields compared to rigid RFID sensor devices.

### 3.2. Commercial Solutions

Due to the great commercial opportunities in the RFID sensing techniques in the future IoT era, some pioneers in the industry including TI, STMicroelectronics, ASM, Farsen, Axzon, and Impinj have also devoted much effort to the research and development in this particular field. Some novel RF-to-DC converter modules, RFID transceivers, and RFID ICs are invented which can be found on the market. Some novel exemplar technical solutions for different applications are found on the market as well.

#### 3.2.1. Promising RFID ICs for Sensor Development

Many RFID ICs were invented as RFID transponders in RFID applications, including both UHF EPC Class-1 G2 and NFC ISO/IEC14443 and ISO/IEC15693, some representatives of which are summarized in Table 2. Most of these ICs integrate RF energy harvesting circuits, internal logic control and memories, and a serial port to allow the read/write capability for an external MCU in order to update the sensor data. Analog-to-Digital Converters (ADCs) are included in some RFID ICs for interfacing with sensor components, such as the MLX90129 and SL13A [86,87]. The model RF430FRL152H from TI integrating a low-power microcontroller MSP430 and a 14-bit digital signal A/D interface has effectively facilitated the further development [88]. SL900A and Magnus-S3 M3D IC have gone further by integrating temperature and capacitive sensors, and integrating temperature, moisture sensors, and proximity sensors, respectively [89]. The integration of built-in sensing modules allows even more convenient development for different applications. The most convenient choices for RFID sensor development are the Rocky100, RF430CL330H and RF430FRL152H, which have RF-to-DC converters and also power output in order to power the external sensors and MCUs. They can be used to develop battery-less RFID sensor devices.

#### 3.2.2. Commercial RFID Sensor Applications

**Figure 10:**
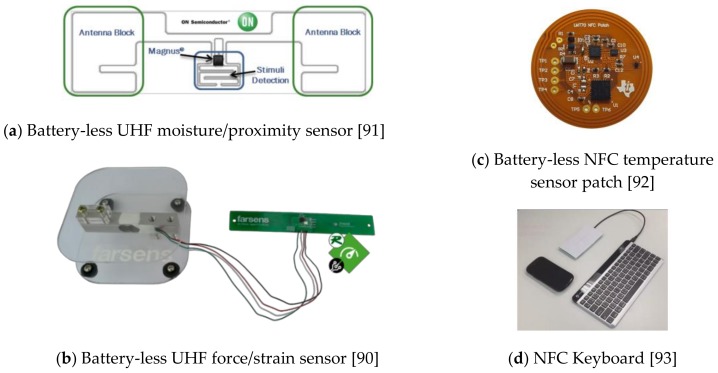
Examples of Commercial RFID Sensor Solutions.

Based on the RFID ICs, there are also sample application modules. Farsens has produced a series of UHF RFID solutions for industrial, agricultural and logistics applications, including battery-less sensor modules, including temperature sensors, ambient light sensors, pressure sensors, magnetic field sensors, humidity/moisture sensors (Figure 10a), force/strain sensors (Figure 10b), and RF field sensors [90]. Similarly, On Semiconductor provides UHF RFID battery-free wireless sensors solutions based on the MagnusS2® Sensor IC, including moisture/proximity and temperature/proximity sensors [91]. In addition, TI has provided some NFC sensing modules based on its RFID IC products, such as a NFC temperature sensor patch (Figure 10c) [92] providing temperature readings to NFC enabled smartphones, and NFC keyboards (Figure 10d) as an alternative to touchscreen keypads for NFC-enabled smartphones and tablets [93]. Novel developments are not limited to the abovementioned examples. Due to the features in wireless power and data transmission, battery-less and light-weight, contactless sensing, the RFID sensing techniques will play an important role in the future IoT market, including consumer electronics, manufacturing, agriculture, medical devices and logistics, etc.

### 3.3. Innovative Applications

**Figure 11:**
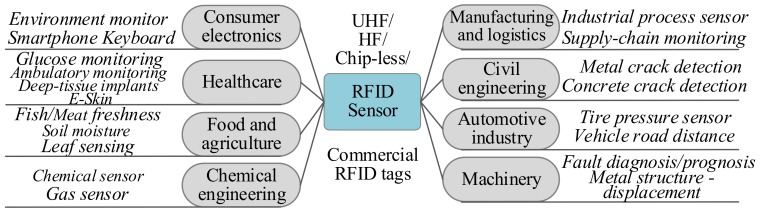
Application Fields and Examples of RFID Sensor Techniques.

RFID sensor techniques, taking advantage of both wireless power and data transfer and object identification, have been a new way of sensing and communication which can now find applications in a wide spectrum of fields. On the one hand, the wireless power and data transfer provides an interface for transmitting sensing data out from the object under test without wires and batteries, which results in substantial miniaturization of the sensor devices. On the other hand, the unique identification code distinguishes the object under tests and integrates the data with its ID directly. In many aspects, these two strengths make RFID sensing devices more competitive than traditional wireless sensor nodes, which need to carry a battery and a wireless module. Therefore, with the novel integration of RFID and lightweight sensor techniques, some measurement tasks which are technically challenging in the past become much more convenient. The fields of application include consumer electronics, healthcare, food and agriculture, chemical engineering, manufacturing and logistics, civil engineering, automotive, machinery, etc., which are summarized with typical examples as shown in Figure 11.

**Figure 12:**
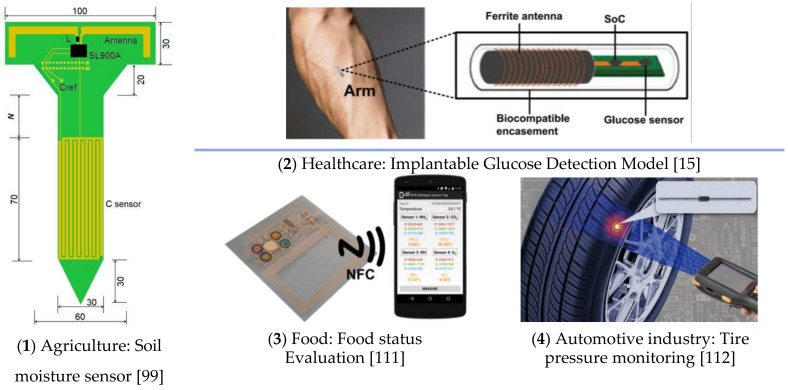
Typical Application of RFID Sensors.

Some novel applications of RFID sensors in the literature regarding the interface protocols, sensing techniques, fabrication process, and sizes are summarized in Table 3 and Figure 12. From Figure 12, it is easy to find that:

(1) RFID sensors of different types, including HF, UHF, and chip-less RFID devices have been widely used and integrated with cutting-edge technologies in different fields in recent years. This has been an effective and cost-efficient means for accessing sensing data in a battery-less, wireless, and passive way, which were significant technical challenges before RFID was invented;

(2) The RFID technique, as a power and communication interface in sensor solutions, is easy to integrate with other sensing modules with different processes, including inkjet printing, CMOS, flexible PCB, etc. Therefore, the functionality is easily extended for different purposes and application scenarios;

(3) The sensor techniques are integrated with RFID in different ways: a. digital sensors with RFID ICs; b. sensitive materials integrated with RFID antennas; c. commercial RFID tags; d. chip-less RFID antenna as a sensor. 

## 4. Technical Challenges

Although continuous technical progresses have been made and many innovative applications of RFID-based sensor techniques are found in the literature, most of the presented work are still in the stage of proof-of-concept demonstration and in-lab test and evaluation. It is hard to find RFID sensor applications on the market besides a few commercial solutions. There is still a big gap between the investigations and practical applications. In summary, the key technical challenges are identified and listed as follows:

### 4.1. Efficiency of RF Frontend Energy Harvesting and Power Conversion

For an RFID tag, the antenna and IC just receive the RF power, store the energy and power the controller to respond to the read/write commands. All the power conversion and logical operations are completed in the RFID IC, which makes energy harvesting efficient, and therefore the reading distance normally reaches as far as 10 m. However, for RFID sensors, the RF energy harvesting front end needs to power the RFID IC, the MCU, and the sensing module. Although all the components are power efficient, the operation logic of the sensors are more complex and time consuming. Therefore, it is still a challenge to power all the components and cover the logic operations with RF energy harvesting only. Especially when the sensors are implanted in the materials under test, the RF signal is attenuated by the ambient materials and the received RF energy can hardly power all the operations, which seriously affects the read/write range of the RFID sensor.

### 4.2. Heterogeneity in the Enabling Techniques

The underlying techniques of RFID sensors show great heterogeneities in antennas, IC functionalities, sensing components, and data protocols, etc. The heterogeneity hinders the techniques from cross-platform integration and standardization. Most designs in the investigations are custom designed for the particular sensing scenarios, including the antennas, ICs, control logic, and data transmission. On the one hand, in order to gain high energy harvesting efficiency, the impedance of the antenna and RFID IC ports should satisfy a conjugate matching. Due to the variations of the impedance in RFID IC ports, the antennas need to be custom-designed for different ICs. For example, the port impedance for Impinj Monza 4 at 915MHz is (11 + j143)Ω [111,112,113], and those for the AMS AG SL900A and Farsens Rocky100 at 915 MHz are (123 − j303)Ω and (52 − j479)Ω, respectively [114,115], which results in a requirement for custom-designed antennas. On the other hand, the sensor data transmission protocols are variables for different applications. UHF sensor solutions may utilize the user ID section to accommodate the sensor data. But there are no widely accepted coding protocols for different sensor categories and measurement accuracies.

### 4.3. Reliability

RFID sensors are mainly attached to the measured object for identification and parameter sensing. However, they are also influenced by certain environmental factors in their actual applications. This not only requires high performance RFID sensors, but also requires RFID sensors to have the ability to cope with harsh environmental features such as high temperature, high pressure, humidity and impact. The metal package also blocks the information transmitted by the RFID sensor, causing the RFID sensor to malfunction. Therefore, in order to obtain stable RFID sensor information, the reliability of RFID sensor devices and antennas under severe conditions still poses great challenges.

The above technical issues have been the dominant obstacles facing the progress of RFID sensors. However, the products of the pioneer companies such as TI, AMS AG, Impinj, Farsens, Axzon, and some innovative applications have paved the way for building blocks for RFID sensor techniques. By overcoming the technical issues in energy harvesting efficiency and heterogeneities, the RFID sensing techniques will play an important role in the future rich sensing IoT world.

## 5. Future Prospectives

RFID sensor techniques will continue to attract interest in a variety of fields in both industry and academia. This section identifies the key research areas of RFID sensor research, focusing on the techniques for improving the sensor performance and the futuristic promising applications. Particular attention has been paid to the IoT applications of RFID sensing techniques, which is considered a revolutionary technology for future information systems.

### 5.1. Research Focuses

The means to improve the performance of RFID sensor techniques such as integration of some new materials and new processes will become a key research area. RFID relay technique for the purpose of extending the reading range of RFID sensors will be of interest as well. In addition, RFID sensor network for wide area and multiple object monitoring taking advantage of the battery-less and light-weight performance can find its place in academic research.

#### 5.1.1. Integration with New Materials and New Processes

New materials and processes will be introduced to RFID sensing techniques in order to improve the performance compared to traditional means, such as graphene dipole antenna on paper substrate UHF RFID [116], graphene nanoflake-printed flexible meandered-line dipole antenna on paper substrate for UHF RFID [117], and RFID passive gas sensors integrating carbon nanotubes [118]. The application of new materials may effectively reduce the cost with acceptable performance. New processes such as CMOS processes, inkjet printing and 3D printing are being introduced to RFID sensor techniques as well. The integration of the new materials and new processes will create new opportunities for RFID sensing techniques, which will become a critical research direction.

#### 5.1.2. Relay Resonator for Extending RFID Reading Distance

Power transmission has been a limit for lossy materials in some applications, such as human tissue implantable sensors and civil structure built-in sensors. A power relay of the sensor system will effectively enhance the functionality and extend the usability of the sensing devices. For instance, a relay resonator was designed for HF RFID food monitoring by Cao and Chung, which has effectively extended the reading distance to 5 cm [34]. It is evident that effective technical solutions for extending the RFID working distance will be of interest for some applications.

#### 5.1.3. RFID Sensor Based Wireless Sensor Network (WSN)

The integration of RFID and WSN can increase their utility in other scientific and engineering fields by exploiting the advantages of both technologies. However, these two technologies have separate research and development areas. As an integration of both, the system architecture, communication protocols, and data presentation are still under investigation and practice [119]. The integration of the advantage of wireless power and sensor data transfer of RFID sensor techniques will create new chances for WSN applications [120].

#### 5.1.4. RFID Sensor Based IoT Applications

Since IoT techniques have spread to a variety of fields, including manufacturing, supply chain, elderly care, agriculture, livestock management, etc., and most of the relevant applications may include RFID tags to identify the product item, human, location, or animal, etc. Normally, the identified items may have their data recorded with different sensing approaches. The integration of sensor interfaces with RFID will significantly promote the efficiency of data accessing for these IoT applications.

### 5.2. Promising IoT Applications

The advantages in sensing and communication of IoT sensor techniques have promised a very big market in the future IoT world. RFID sensor techniques will revolutionize the traditional non-sensing RFID based application, especially in the IoT applications. 

#### 5.2.1. Product Lifecycle Management (PLM) in Manufacturing Industry

RFID tags are implemented in manufacturing industry and supply chain for product identification in different stages of the production. By integrating the sensor techniques with RFID, the key parameters of products in these stages can be observed and the product information at all stages can be used for further analysis in order to optimize the product design and the production process. RFID sensor can potentially revolutionize the efficiency and management of manufacturing and supply chain. 

#### 5.2.2. Continuous Monitoring of Human Physical Characteristics

For medical care and elderly care, RFID sensors are an effective sensing and data transmission interface, which has already been an interesting research topic. RFID sensor networks for the monitoring of human physical characteristics will be an effective way for data collection and transmission, especially for some vital signs of chronic diseases. 

#### 5.2.3. RFID Sensors in Smart Logistics

Current logistics applications may include RFID tags in order to identify the product items at the key nodes of products to track its information. Sensor-enabled RFID could monitor the quality of products through the whole logistics process, which may extend the products monitored from traditional things to fresh food and live plants, etc.

#### 5.2.4. RFID Sensors in Smart Agriculture

Future agriculture may become highly automated and informatized. The integration of sensing techniques and RFID has been a solution for monitoring some key parameters, such as humidity, temperature, and light strength of different identifiable positions using a drone carrying a RFID reader. The collected data can be used to predict the health status and grade of maturity of plants. By taking advantages of RFID for sensing purpose in different fields, RFID sensor techniques may be of particular interest in the above fields. Compared to traditional RFID-based applications, the RFID sensors featuring wireless power and data transfer, real-time and continuous data collection could potentially revolutionize traditional applications. Due to their unique advantages, RFID sensors will be a competitive choice for various measurement applications.

## 6. Summary

The rapid technical progress and widespread application of RFID sensing techniques have produced novel solutions in different fields of applications, which have very promising prospective for future IoT rich sensing applications. This investigation identifies the gaps between in-lab studies and practical applications and provides a thorough overview of the underlying theories, technical progress, and real-world application examples covering different application scenarios.

Based on the survey of the state-of-the-art studies, it is found that: (1) RFID sensors are an effective and cost-efficient means for accessing sensing data in a battery-less, wireless, and passive way, which were technical challenges before RFID was invented, and they will play an important role in the future; (2) RFID sensor technology integrates sensing techniques and RFID with many cutting-edge techniques, including CMOS, flexible PCB, inkjet-printed electronics, etc. The critical technical challenges lie in: (1) Efficiency of the RF frontend energy harvesting and power conversion is a limitation for some miniature sensor applications; (2) the heterogeneity in antenna, RFID ICs, the way of applications, and sensor data reading protocols. An industrial level standardization or guidelines for RFID sensing techniques are expected to simplify the applications.

The RFID sensing techniques will continuously attract research efforts in both industry and the academia in the forthcoming IoT era, when the sensing and communication become the basis of the information infrastructure. The RFID sensor technology may find more applications in biomedical areas for implantation in the human body, in civil engineering to be integrated in civil structures for health monitoring, in food engineering for low-cost quality monitoring. Due to the rapid progress of the relevant techniques in the manufacturing industry, such as smart robotics and smart controllers, RFID sensing techniques are a promising opportunity for Product Lifecycle Management (PLM), which could potentially build a comprehensive information link between each life stage of products, including raw materials, production processes, logistics, usage, and disposal. The key parameters of products in some life stages can be observed and the data can be collected for further analysis. Based on the related investigations, we can draw the conclusion that in strategic fields such as manufacturing, healthcare, automotive industry and transport, and energy saving, RFID sensor techniques will play an important role due to the strengths in wireless data transmission, battery-less, power-efficiency and cost-efficiency and the extreme power constraints in Internet of Things applications are driving the emergence of new devices and innovative solutions.

## Figures and Tables

**Table 1:** RFID Antennas.

Novel Antenna	Carrier Frequency	Size	Reading Range	Investigators
metallic surface mountable antennas	UHF	30 × 18× 3.2mm^3^	1.5 m	Chen [39]
	UHF	106 × 44 × 5mm^3^	N/A	Xu et al. [40]
	UHF	104 × 31 × 7.6mm^3^	14.6m	Hamani et al. [41]
	UHF	41.5 × 55 × 3 mm^3^	Metal: 6.1m Dielectrics: 14.1m	Li et al. [42]
3D antennas	UHF	3 × 3 × 3 cm^3^	N/A	Kruesi et al. [43]
	UHF	50 × 50 × 50 mm^3^	N/A	Jin et al. [44]
	UHF	2 × 2 × 1.2 cm^3^	N/A	Galehdar et al. [45]
multi-band antennas	UHF-HF	85 × 54 × 0.8 mm^3^	N/A	Ma et al. [46]
	UHF-MW	L: 19 cm	N/A	Keyrouz et al. [47]
	UHF-MW	30 × 30 mm^2^	N/A	Li et al. [48]
omnidirectional and directional antennas	UHF	866 MHz: 98.7 × 14.2 mm^2^ 915 MHz: 88 × 14.2 mm^2^ 953 MHz: 84 × 14.2 mm^2^	N/A	Tang et al. [49]
	UHF	43 × 43 mm^2^	9.5 m	Liu et al. [50]
	UHF Near-Field	R: 80 mm	120 mm	Zeng et al. [51]

**Table 2:** RFID ICs.

Manufacturer	IC Model	RF Band/ Protocols	RF Sensitivity	Digital Interfaces	Power Output	Packaging
Farsens	Rocky100	UHF/EPC Class-1 G2	−13 dBm	SPI	1.2 V~3.0 V	QFN-16: 4 × 4 mm
Ramtron	WM72016	UHF/EPC Class-1 G2	< −6 dBm	DSPI	N/A	UDFN-8: 3 × 3 mm
AMS	SL900A	UHF/EPC Class-3	−7 dBm	SPI	N/A	QFN-16: 5 × 5 mm
Axzon (formerly RFMicron)	Magnus-S3 M3D IC	UHF EPC Class 1 G2 and ISO/IEC18000	N/A	N/A	N/A	QFN
Impinj	Monza X-2K/X-8K	UHF/EPC Class-1 G2	−17 dBm	I2C	N/A	XQFN-8L:1.65 × 1.65 mm
AMS	AS3953A	HF NFC/ISO14443A-4	N/A	SPI	N/A	WLCSP-10: 3 × 3 mm
AMS	SL13A	HF NFC/ISO 15693	N/A	SPI	N/A	QFN-16LD: 5 × 5 mm
TI	RF430CL330H	HF NFC/ISO14443B	N/A	SPI/I2C	N/A	TSSOP-14 VQFN-16
TI	RF430FRL152H	HF NFC/ISO/IEC 15693	N/A	SPI/I2C	N/A	VQFN-24: 4 × 4 mm
Melexis	MLX90129	HF NFC/ISO/IEC 15693	N/A	SPI	2.8 V~3.2 V	TSSOP-20
Silicon Craft	SIC4310/ 4340/4341	ISO 14443A	N/A	UART	N/A	QFN-16: 3 × 3 mm
NXP	NT3H1101/1201	ISO 14443-3	N/A	I2C	N/A	SOT902-3: 1.6 × 1.6 × 0.6 mm
NXP	SL3ICS1002/1202	UHF/EPC Class-1 G2	N/A	/	N/A	TSSOP-8: 4.9 × 3 mm
ST Micro-electronics	M24LR0xE-R/ST25DV-I2C	HF NFC/ISO15693	N/A	I2C	N/A	SO8N: 4.9 × 6 mm TSSOP8: 3 × 6.4 mmUFDFN8: 2 × 3 mm
On Semiconductor	N24RF64	HF NFC/ISO15693	N/A	I2C	N/A	SOIC-8: 6 × 5 mm TSSOP-8: 6.4 × 3 mm

**Table 3:** Novel Applications of RFID Sensors.

Functions	Interface Protocols	Sensing Techniques	Fabrication Process	Size	Investigators
Humidity monitoring	Chip-less RFID	Artificial impedance surface	Inkjet Printed	77 × 58 × 43 cm^3^	Borgese et al., 2017 [78]
Glucose monitoring	HF NFC ISO15693	Potentiostat	0.13 μm CMOS	1.2 × 2 mm	Xiao et al., 2015 [15]
Ambulatory monitoring	UHF RFID	Accelerometer	PCB circuit	N/A	Wickramasinghe et al., 2015 [94]
Implanted sensors	RFID (100 MHz)	Pressure sensor	Copper tape made	mm-sized	Moradi et al., 2017 [95]
E-Skin sensor	HF	Coil antennas	Flexible PCB circuit	134.4 × 18.2 mm	Baek et al., 2018 [96]
Fish freshness	HF NFC	Resistive sensor and humidity sensors	Flexible PCB circuit	N/A	Smits, et al., 2012 [97]
Meat freshness	UHF	Temperature, humidity, gas	Assembled with modules	N/A	Eom et al., 2014 [98]
Soil moisture	UHF (SL900A)	Capacitive sensor	PCB circuit	100 × 180 mm	Pichorim et al., 2018 [99]
Chemical sensor	UHF	Sensitive coating materials	Flexible PCB circuit	55 × 45 mm	Manzari et al., 2014 [100]
Industrial process parameters	UHF RFID	Vibration, light, temperature, humidity	PCB circuit	80 × 120 mm	Petrov, et al., 2019 [101]
Supply chain monitoring	HF NFC ISO15693	Critical temperature indicator (CTI)	PCB circuit	Sensor: 35 × 10 × 2 mm Tag: not given	Lorite et al., 2017 [102]
Strain	UHF	Resistive strain gauge	PCB circuit	150 × 120 mm	DiGiampaolo et al., 2017 [103]
Metal crack detection	Chip-less RFID	Microstrip patch antenna resonator	PCB antenna	35 × 15 mm	Marindra et al., 2018 [104]
Concrete crack	UHF RFID	Couplet of RFID tags	RFID tag designed	N/A	Caizzone and DiGiampaolo, 2015 [105]
Corrosion in concrete	HF NFC ISO15693	Three-electrode potentionstat	PCB circuit	11.8 × 4 × 5.6 cm	Leon-Salas and Halmen, 2016 [106]
Bicycle tire pressure measurement	HF NFC ISO14443	Capacitive sensor	130 nm CMOS	5.76 mm^2^	Kollegger et al., 2017 [107]
Vehicle road distancer measurement	UHF RFID	Distance	Commercial tags	N/A	Huo et al., 2014 [108]
Fault diagnosis & prognosis	UHF (Monza X-8K)	Accelerometer	PCB circuit	Long: 12 mm	Wang et al., 2017 [109]
Metal structure displacement	UHF RFID	Deformation sensor	Custom designed	72 × 37 mm	Kuhn et al., 2018 [110]

## References

[1] Al-Fuqaha A., Guizani M., Mohammadi M., Aledhari M., Ayyash M. (2015). Internet of things: A survey on enabling technologies, protocols, and applications. IEEE Commun. Surv. Tutor..

[2] Xu L.D., He W., Li S. (2014). Internet of things in industries: A survey. IEEE Trans. Ind. Inform..

[3] Want R. (2006). An introduction to RFID technology. IEEE Pervas. Comput..

[4] Welbourne E., Battle L., Cole G., Gould K., Rector K., Raymer S., Balazinska M., Borriello G. (2009). Building the internet of things using RFID: the RFID ecosystem experience. IEEE Internet Comput..

[5] Wang T., He Y., Shi T., Li B. (2019). Transformer incipient hybrid fault diagnosis based on solar-powered RFID sensor and optimized DBN approach. IEEE Access.

[6] Kaneko M., Hu W., Hayashi K., Sakai H. (2014). Compressed sensing-based tag identification protocol for a passive RFID system. IEEE Commun. Lett..

[7] Zeng L., Grau D., Xiao Y. (2015). Assessing the feasibility of passive and BAP RFID communications on construction site scenarios. IEEE Syst. J..

[8] Zhang J., Tian G.Y., Marindra A.M.J., Sunny A.I., Zhao A.B. (2017). A review of passive RFID tag antenna-based sensors and systems for structure health monitoring applications. Sensors.

[9] Oprea A., Bârsan N., Weimar U., Bauersfeld M.L., Ebling D., Wöllenstein J. (2008). Capacitive humidity sensors on flexible RFID labels. Sensor. Actuat. B-Chem..

[10] Occhiuzzi C., Paggi C., Marrocco G. (2011). Passive RFID strain-sensor based on meander-line antennas. IEEE Trans. Antennas Propag..

[11] Rennane A., Abdelnour A., Kaddour D., Touhami R., Tedjini S. (2018). Design of passive UHF RFID sensor on flexible foil for sports balls pressure monitoring. IET Microw. Antennas Propag..

[12] Zhang J., Tian G.Y., Zhao A.B. (2017). Passive RFID sensor systems for crack detection & characterization. NDT&E Int..

[13] Caizzone S., DiGiampaolo E. Passive RFID Deformation Sensor for Concrete Structures. Proceedings of the 2014 IEEE RFID Technology and Applications Conference (RFID-TA).

[14] Zarifi M.H., Deif S., Daneshmand M. (2017). Wireless passive RFID sensor for pipeline integrity monitoring. Sens. Actuat. A-Phys..

[15] Xiao Z., Tan X., Chen X., Chen S., Zhang Z., Zhang H., Wang J., Huang Y., Zhang P., Zheng L. (2015). An implantable RFID sensor tag toward continuous glucose monitoring. IEEE J. Biomed. Health.

[16] Caldara M., Nodari B., Re V., Bonandrini B. (2014). Miniaturized and low-power blood pressure telemetry system with RFID interface. Procedia Eng..

[17] Turner M., Naber J. The Development of a RFID Based Mixed Signal ASIC for the Wireless Measurement of Intraocular Pressure. Proceedings of the 2010 18th Biennial University/Government/Industry Micro/Nano Symposium.

[18] Caccami M.C., Mulla M.Y.S., Occhiuzzi C., Di Natale C., Marrocco G. (2018). Design and experimentation of a batteryless on-skin RFID graphene-oxide sensor for the monitoring and discrimination of breath anomalies. IEEE Sens. J..

[19] Lazaro A., Boada M., Villarino R., Girbau D. (2019). Color measurement and analysis of fruit with a battery-less NFC sensor. Sensors.

[20] Lumpkins W. (2013). Nikola Tesla’s dream realized: Wireless power energy harvesting. IEEE Consum. Electron. Mag..

[21] Brown W.C. (1984). The history of power transmission by radio waves. IEEE Trans. Microw. Theory.

[22] Hemour S., Wu K. (2014). Radio-frequency rectifier for electromagnetic energy harvesting: Development path and future outlook. Proc. IEEE.

[23] Zargham M., Gulak P.G. (2012). Maximum achievable efficiency in near-field coupled power-transfer systems. IEEE Trans. Biomed. Circuits Syst..

[24] De Venuto D., Rabaey J. (2014). RFID transceiver for wireless powering brain implanted microelectrodes and backscattered neural data collection. Microelectron. J..

[25] Shan C.Y., Shan Y.F., Yao L. (2015). Radio Frequency Identification (RFID) Principles and Applications.

[26] Cao X.T., Chung W.Y. (2019). An enhanced multiplication of RF energy harvesting efficiency using relay resonator for food monitoring. Sensors.

[27] Kim S., Georgiadis A., Tentzeris M. (2018). Design of inkjet-printed RFID-based sensor on paper: Single-and dual-tag sensor topologies. Sensors.

[28] AN 1629 UHF RFID Label Antenna Design. https://www.nxp.com/docs/en/application-note/AN162910.pdf.

[29] Nikitin P.V., Rao K.V.S., Lazar S. An Overview of Near Field UHF RFID. Proceedings of the 2007 IEEE International Conference on RFID.

[30] Herrojo C., Muela F.J., Mata-Contreras J., Paredes F., Martín F. (2019). High-density microwave encoders for motion control and near-field chipless-RFID. IEEE Sens. J..

[31] Feng Y., Xie L., Chen Q., Zheng L.R. (2014). Low-cost printed chipless RFID humidity sensor tag for intelligent packaging. IEEE Sens. J..

[32] Athauda T., Karmakar N.C. (2019). The realisation of chipless RFID resonator for multiple physical parameter sensing. IEEE Internet Things.

[33] Shen Y., Law C.L. (2012). A low-cost UWB-RFID system utilizing compact circularly polarized chipless tags. IEEE Antennas Wirel. Propag. Lett..

[34] Kim S., Vyas R., Bito J., Niotaki K., Collado A., Georgiadis A., Tentzeris M.M. (2014). Ambient RF energy-harvesting technologies for self-sustainable standalone wireless sensor platforms. Proc. IEEE.

[35] Bi S., Ho C.K., Zhang R. (2015). Wireless powered communication: Opportunities and challenges. IEEE Commun. Mag..

[36] Ng D.W.K., Lo E.S., Schober R. (2013). Wireless information and power transfer: Energy efficiency optimization in OFDMA systems. IEEE Trans. Wirel. Commun..

[37] Gope P., Amin R., Islam S.K.H., Kumar N., Bhalla V.K. (2018). Lightweight and privacy-preserving RFID authentication scheme for distributed IoT infrastructure with secure localization services for smart city environment. Future Gener. Comput. Syst..

[38] Deng X.Y., Wang Y., He Y.J. (2016). Passive RFID Electronic Label Wireless Theory and Engineering.

[39] Chen S.L. (2009). A miniature RFID tag antenna design for metallic objects application. IEEE Antennas Wirel. Propag. Lett..

[40] Xu L., Tian L., Hu B. A Novel Broadband UHF RFID Tag Antenna Mountable on Metallic Surface. Proceedings of the 2007 International Conference on Wireless Communications, Networking and Mobile Computing.

[41] Hamani A., Yagoub M.C.E., Vuong T.P., Touhami R. (2016). A novel broadband antenna design for UHF RFID tags on metallic surface environments. IEEE Antennas Wirel. Propag. Lett..

[42] Li H., Zhu J., Yu Y. (2017). Compact single-layer RFID tag antenna tolerant to background materials. IEEE Access.

[43] Kruesi C.M., Vyas R.J., Tentzeris M.M. (2009). Design and development of a novel 3-D cubic antenna for wireless sensor networks (WSNs) and RFID applications. IEEE Trans. Antennas Propag..

[44] Jin X., Dang X., Yang F. Compact 3-D cubic loop antennas with omnidirectional patterns. Proceedings of the 2014 IEEE Antennas and Propagation Society International Symposium (APSURSI).

[45] Galehdar A., Thiel D.V., O’Keefe S.G. (2009). Design methods for 3D RFID antennas located on a conducting ground plane. IEEE Trans. Antennas Propag..

[46] Ma Z.L., Jiang L.J., Xi J., Ye T.T. (2012). A single-layer compact HF-UHF dual-band RFID tag antenna. IEEE Antennas Wirel. Propag. Lett..

[47] Keyrouz S., Visser H.J., Tijhuis A.G. Multi-Band Simultaneous Radio Frequency Energy Harvesting. Proceedings of the 2013 7th European Conference on Antennas and Propagation (EuCAP).

[48] Li H.H., Mou X.Q., Ji Z., Yu H., Li Y., Jiang L. (2011). Miniature RFID tri-band CPW-fed antenna optimised using ISPO algorithm. Electron. Lett..

[49] Tang Z.J., He Y.G., Wang Y. (2011). Broadband UHF RFID tag antenna with quasi-isotropic radiation performance. AEU-Int. J. Electron. C..

[50] Liu Q., Yu Y., He S. (2013). Capacitively loaded, inductively coupled fed loop antenna with an omnidirectional radiation pattern for UHF RFID tags. IEEE Antennas Wirel. Propag. Lett..

[51] Zeng Y., Chen Z.N., Qing X., Jin J.M. (2018). A directional, closely spaced zero-phase-shift-line loop array for UHF near-field RFID reader antennas. IEEE Trans. Antennas Propag..

[52] De Vita G., Iannaccone G. (2005). Design criteria for the RF section of UHF and microwave passive RFID transponders. IEEE Trans. Microw. Theory.

[53] Yi J., Ki W.H., Tsui C.Y. (2007). Analysis and design strategy of UHF micro-power CMOS rectifiers for micro-sensor and RFID applications. IEEE Trans. Circuits Syst. I.

[54] Liu D.S., Zuo X.C., Dai K., Li S.Z., Hui X.M., Liu Y., Tong Q.L. (2010). New design of RF rectifier for passive UHF RFID transponders. Microelectron. J..

[55] Ghovanloo M., Atluri S. (2008). An integrated full-wave CMOS rectifier with built-in back telemetry for RFID and implantable biomedical applications. IEEE Trans. Circuits Syst. I.

[56] Xu H., Ortmanns M. (2011). A temperature and process compensated ultralow-voltage rectifier in standard threshold cmos for energy-harvesting applications. IEEE Trans. Circuits Syst. II.

[57] Galup-Montoro C., Schneider M.C., Machado M.B. (2012). Ultra-low-voltage operation of CMOS analog circuits: amplifiers, oscillators, and rectifiers. IEEE Trans. Circuits Syst. II.

[58] Theilmann P.T., Presti C.D., Kelly D.J., Asbeck P.M. (2012). A*μ*W complementary bridge rectifier with near zero turn-on voltage in SOS CMOS for wireless power supplies. IEEE Trans. Circuits Syst. I.

[59] Hwang Y.S., Hwang B.H., Lin H.C., Chen J.J. (2011). PLL-based contactless energy transfer analog FSK demodulator using high-efficiency rectifier. IEEE Trans. Ind. Electron..

[60] Nguyen T.T., Feng T., Häfliger P., Chakrabartty S. (2014). Hybrid CMOS rectifier based on synergistic RF-piezoelectric energy scavenging. IEEE Trans. Circuits Syst. I.

[61] Chouhan S.S., Halonen K. (2015). Threshold voltage compensation scheme for RF-to-DC converter used in RFID applications. Electron. Lett..

[62] Tran L.G., Cha H.K., Park W.T. (2017). RF power harvesting: A review on designing methodologies and applications. Micro Nano Syst. Lett..

[63] Wei P., Che W., Bi Z., Wei C., Na Y., Qiang L., Hao M. (2011). High-efficiency differential RF front-end for a Gen2 RFID tag. IEEE Trans. Circuits Syst. II.

[64] Bakhtiar A.S., Jalali M.S., Mirabbasi S. A High-Efficiency CMOS Rectifier for Low-Power RFID Tags. Proceedings of the 2010 IEEE International Conference on RFID (IEEE RFID 2010).

[65] Ye S., Fan J. A High Efficiency CMOS Rectifier Circuit for 900MHz Passive RFID Tags. Proceedings of the 2010 Second Pacific-Asia Conference on Circuits, Communications and System.

[66] Ouda M.H., Khalil W., Salama K.N. (2016). Wide-range adaptive RF-to-DC power converter for UHF RFIDs. IEEE Microw. Wirel. Compon. Lett..

[67] Raben H., Borg J., Johansson J. (2012). A model for MOS diodes with *V_th_* cancellation in RFID rectifiers. IEEE Trans. Circuits Syst. II.

[68] Hwang Y.S., Lei C.C., Yang Y.W., Chen J.J., Yu C.C. (2014). A 13.56-MHz low-voltage and low-control-loss RF-DC rectifier utilizing a reducing reverse loss technique. IEEE Trans. Power Electron..

[69] Colella R., Pasca M., Catarinucci L., Tarricone L., D’Amico S. (2016). High-sensitivity CMOS RF-DC converter in HF RFID Band. IEEE Microw. Wirel. Compon. Lett..

[70] Yuan J.S., Bi Y. (2015). Process and temperature robust voltage multiplier design for RF energy harvesting. Microelectron. Reliab..

[71] Chouhan S.S., Nurmi M., Halonen K. (2016). Efficiency enhanced voltage multiplier circuit for RF energy harvesting. Microelectron. J..

[72] Kim S., Mariotti C., Alimenti F., Mezzanotte P., Georgiadis A., Collado A., Roselli L., Tentzeris M.M. (2013). No battery required: Perpetual RFID-enabled wireless sensors for cognitive intelligence applications. IEEE Microw. Mag..

[73] Son H.W. (2008). Design of RFID tag antenna for metallic surfaces using lossy substrate. Electron. Lett..

[74] Khan S., Lorenzelli L., Dahiya R.S. (2014). Technologies for printing sensors and electronics over large flexible substrates: a review. IEEE Sens. J..

[75] Salmerón J.F., Molina-Lopez F., Rivadeneyra A., Quintero A.V., Capitán-Vallvey L.F., de Rooij N.F., Ozáez J.B., Briand D., Palma A.J. (2014). Design and development of sensing RFID Tags on flexible foil compatible with EPC Gen 2. IEEE Sens. J..

[76] Kim S., Kawahara Y., Georgiadis A., Collado A., Tentzeris M.M. (2014). Low-cost inkjet-printed fully passive RFID tags for calibration-free capacitive/haptic sensor applications. IEEE Sens. J..

[77] Sharif A., Ouyang J., Yang F., Chattha H.T., Imran M.A., Alomainy A., Abbasi Q.H. (2019). Low-cost inkjet-printed UHF RFID tag-based system for internet of things applications using characteristic modes. IEEE Internet Things.

[78] Borgese M., Dicandia F.A., Costa F., Genovesi S., Manara G. (2017). An inkjet printed chipless RFID sensor for wireless humidity monitoring. IEEE Sens. J..

[79] Salmerón J.F., Rivadeneyra A., Agudo-Acemel M., Capitán-Vallvey L.F., Banqueri J., Carvajal M.A., Palma A.J. (2014). Printed single-chip UHF passive radio frequency identification tags with sensing capability. Sensor. Actuat. A-Phys..

[80] Kallmayer C., Pisarek R., Neudeck A., Cichos S., Gimpel S., Aschenbrenner R., Reichl H. New Assembly Technologies for Textile Transponder Systems. Proceedings of the Electronic Components and Technology Conference.

[81] Vieroth R., Kallmayer C., Aschenbrenner R., Reichl H. A New Package for Textile Integrated RFID Tags. Proceedings of the 2009 11th Electronics Packaging Technology Conference.

[82] Alonso-Gonzalez L., Ver-Hoeye S., Vazquez-Antuna C., Fernández-García M., Las-Heras Andrés F. (2019). Multifunctional fully textile-integrated RFID tag to revolutionize the internet of things in clothing [wireless corner]. IEEE Antennas Propag. Mag..

[83] Alonso-González L., Ver-Hoeye S., Fernández-García M., Andrés F.L.H. (2018). Broadband flexible fully textile-integrated bandstop frequency selective surface. IEEE Trans. Antennas Propag..

[84] Alonso-González L., Ver-Hoeye S., Fernández-García M., Álvarez-López Y., Vázquez-Antuña C., Andrés F.L.H. (2018). Fully textile-integrated microstrip-fed slot antenna for dedicated short-range communications. IEEE Trans. Antennas Propag..

[85] Alonso-González L., Ver-Hoeye S., Vázquez-Antuña C., Fernández-García M., Andrés F.L.H. (2017). On the techniques to develop millimeter-wave textile integrated waveguides using rigid warp threads. IEEE Trans. Microw. Theory.

[86] Download datasheet for CSA-1VG. https://www.melexis.com/en/documents/documentation/datasheets/datasheet-csa-1vg.

[87] SL13A Smart Sensory Tag Chip for Unique Identification, Monitoring and Data Logging. http://www.mouser.com/ds/2/588/AMS_SL13A_Datasheet_EN_v4-371531.pdf.

[88] RF430FRL15xH NFC ISO 15693 Sensor Transponder. http://www.ti.com/lit/ds/symlink/rf430frl152h.pdf.

[89] RFM3300-D Magnus®-S3 M3D Passive Sensor, IC. https://axzon.com/rfm3300-d-magnus-s3-m3d-passive-sensor-ic/.

[90] EPC C1G2 Batteryless Load Sensor. http://www.farsens.com/wp-content/uploads/2018/06/DS-EVAL01-ZYGOS-RM-V04.pdf.

[91] Battery Free Wireless Sensor. http://www.ebvnews.ru/doc16/SPS1M002-D.pdf.

[92] Passive NFC Temperature Patch Reference Design. http://www.ti.com.cn/cn/lit/ug/tidubt8/tidubt8.pdf.

[93] Battery-Less Near Field Communication (NFC) Keyboard. http://www.ti.com.cn/cn/lit/ug/tidu398/tidu398.pdf.

[94] Wickramasinghe A., Ranasinghe D.C. (2015). Ambulatory monitoring using passive computational RFID sensors. IEEE Sens. J..

[95] Moradi E., Sydänheimo L., Bova G.S., Ukkonen L. (2017). Measurement of wireless power transfer to deep-tissue RFID-based implants using wireless repeater node. IEEE Antennas Wirel. Propag..

[96] Baek J.J., Kim S.W., Park K.H., Jeong M.J., Kim Y.T. (2018). Design and performance evaluation of 13.56-MHz passive RFID for e-skin sensor application. IEEE Microw. Wirel. Compon..

[97] Smits E., Schram J., Nagelkerke M., Kusters R.H.L., Heck G.V., Acht V.V., Koetse M.M., Brand J.V.D., Gelinck G.H., Schoo H.F.M. Development of Printed RFID Sensor Tags for Smart Food Packaging. Proceedings of the 14th International Meeting on Chemical Sensors.

[98] Eom K.H., Hyun K.H., Lin S., Kim J.W. (2014). The meat freshness monitoring system using the smart RFID tag. Int. J. Distrib. Sens. Netw..

[99] Pichorim S., Gomes N., Batchelor J. (2018). Two solutions of soil moisture sensing with RFID for landslide monitoring. Sensors.

[100] Manzari S., Catini A., Pomarico G., Natale C.D., Marrocco G. (2014). Development of an UHF RFID chemical sensor array for battery-less ambient sensing. IEEE Sens. J..

[101] Petrov D., Schmidt M., Hilleringmann U., Hedayat C., Otto T. RFID Based Sensor Platform for Industry 4.0 Application. Proceedings of the Smart Systems Integration, 13th International Conference and Exhibition on Integration Issues of Miniaturized Systems.

[102] Lorite G.S., Selkälä T., Sipola T., Palenzuela J., Jubete E., Viñuales A., Cabañero G., Grande H.J., Tuominen J., Uusitalo S. (2017). Novel, smart and RFID assisted critical temperature indicator for supply chain monitoring. J. Food Eng..

[103] DiGiampaolo E., DiCarlofelice A., Gregori A. (2016). An RFID-enabled wireless strain gauge sensor for static and dynamic structural monitoring. IEEE Sens. J..

[104] Marindra A.M.J., Tian G.Y. (2018). Chipless RFID sensor tag for metal crack detection and characterization. IEEE Trans. Microw. Theory.

[105] Caizzone S., DiGiampaolo E. (2015). Wireless passive RFID crack width sensor for structural health monitoring. IEEE Sens. J..

[106] Leon-Salas W.D., Halmen C. (2015). A RFID sensor for corrosion monitoring in concrete. IEEE Sens. J..

[107] Kollegger C., Greiner P., Steffan C., Wiessflecker M., Froehlich H., Kautzsch T., Holweg G., Deutschmann B. A System-on-Chip NFC Bicycle Tire Pressure Measurement System. Proceedings of the 2017 IEEE 60th International Midwest Symposium on Circuits and Systems (MWSCAS).

[108] Huo Y., Lu Y., Cheng W., Jing T. Vehicle Road Distance Measurement and Maintenance in RFID Systems on Roads. Proceedings of the 2014 International Conference on Connected Vehicles and Expo (ICCVE).

[109] Wang T., He Y., Luo Q., Deng F., Zhang C. (2017). Self-powered RFID sensor tag for fault diagnosis and prognosis of transformer winding. IEEE Sens. J..

[110] Kuhn M.F., Breier G.P., Clarke T.G.R. (2018). Passive Wireless Sensor for Displacement Monitoring in Metal Structures. IEEE Lat. Am. Trans..

[111] Escobedo P., Erenas M.M., Lopez-Ruiz N., Carvajal M.A., Gonzalez-Chocano S., de Orbe-Payá I., Capitán-Valley L.F., Palma A.J., Martínez-Olmos A. (2017). Flexible passive near field communication tag for multigas sensing. Anal. Chem..

[112] RFID Test Tire Pressure. http://www.chinatiredealer.com/news/show-5089.html.

[113] Monza 4 Tag Chip Datasheet. https://support.impinj.com/hc/en-us/articles/202756908-Monza-4-RFID-Tag-Chip-Datasheet.

[114] SL900A EPC Class 3 Sensory Tag Chip–for Automatic Data Logging. https://ams.com/documents/20143/36005/SL900A_DS000294_5-00.pdf/d399f354-b0b6-146f-6e98-b124826bd737.

[115] Rocky100 datasheet. http://www.farsens.com/wp-content/uploads/2017/12/DS-ROCKY100-V04.pdf.

[116] Kopyt P., Salski B., Olszewska-Placha M., Janczak D., Sloma M., Kurkus T., Jakubowska M., Gwarek W. (2016). Graphene-based dipole antenna for a UHF RFID tag. IEEE Trans. Antennas Propag..

[117] Leng T., Huang X., Chang K.H., Chen J.C., Abdalla M.A., Hu Z. (2016). Graphene nanoflakes printed flexible meandered-line dipole antenna on paper substrate for low-cost RFID and sensing applications. IEEE Antennas Wirel. Propag..

[118] Occhiuzzi C., Rida A., Marrocco G., Tentzeris M. (2011). RFID passive gas sensor integrating carbon nanotubes. IEEE Trans. Microw. Theory.

[119] Mitrokotsa A., Douligeris C. (2009). Integrated RFID and sensor networks: Architectures and applications. RFID Sens. Netw..

[120] Yang H., Yang S.H. (2007). RFID sensor network architectures to integrate RFID, sensor and WSN. Meas. Control.

